# Association between Genetic Polymorphisms of DNA Repair Genes and Chromosomal Damage for 1,3-Butadiene-Exposed Workers in a Matched Study in China

**DOI:** 10.1155/2015/234675

**Published:** 2015-08-03

**Authors:** Menglong Xiang, Lei Sun, Xiaomei Dong, Huan Yang, Wen-bin Liu, Niya Zhou, Xue Han, Ziyuan Zhou, Zhihong Cui, Jing-yi Liu, Jia Cao, Lin Ao

**Affiliations:** ^1^Department of Environmental Hygiene, College of Preventive Medicine, Third Military Medical University, Chongqing 400038, China; ^2^Institute of Toxicology, College of Preventive Medicine, Third Military Medical University, Chongqing 400038, China

## Abstract

The aim of the study was to examine the association between polymorphisms of DNA repair genes and chromosomal damage of 1,3-butadiene- (BD-) exposed workers. The study was conducted in 45 pairs of occupationally exposed workers in a BD product workshop and matched control workers in an administrative office and a circulatory water workshop in China. Newly developed biomarkers (micronuclei, MNi; nucleoplasmic bridges, NPBs; nuclear buds, NBUDs) in the cytokinesis-blocked micronucleus (CBMN) cytome assay were adopted to detect chromosomal damage. PCR and PCR-restriction fragment length polymorphism (RFLP) are adopted to analyze polymorphisms of DNA repair genes, such as X-ray repair cross-complementing Group 1 (*XRCC1*), O_6_-methylguanine-DNA methyltransferase (*MGMT*), poly (adenosine diphosphate-ribose) polymerases (*ADPRT*), and apurinic/apyrimidinic endonucleases (*APE1*). The BD-exposed workers exhibited increased frequencies of MNi and NPBs when compared to subjects in the control group. The results also show that the BD-exposed workers carrying *XRCC1* diplotypes *TCGA-CCGG* (4.25 ± 2.06‰) (FR = 2.10, 95% CI: 1.03–4.28) and *TCGG-TCGA* (5.80 ± 3.56‰) (FR = 2.75, 95% CI: 0.76–2.65) had statistically higher NBUD frequencies than those who carried diplotype *TCGG-TCGG* (1.89 ± 1.27‰). Our study suggests that polymorphisms of *XRCC1* gene may influence chromosomal damage in BD-exposed workers.

## 1. Introduction

1,3-Butadiene (BD), a Group 1 carcinogen as classified by IARC in 2008 [1], is widely used as an industrial chemical and is also present in autoemission and tobacco smoke [2]. The carcinogenicity of BD toward rodent animals was realized early [3]. Meanwhile, a series of epidemiological studies concerning North American BD-exposed workers found associations with leukemia [4]. Hence, there is a critical need to identify the early events and factors that are a potential for predicting health effects of BD exposure. Since the major metabolites of BD have been proved to be mutagenic carcinogens [3], the research on the mutagenicity of BD provided by molecular epidemiological studies may offer useful insights.

However, the results of human molecular epidemiological studies on BD have been mixed. In terms of common genotoxic endpoints, only a few studies have yielded positive results. For example, one group studied the population in Texas in the US and reported significantly elevated frequencies of hypoxanthine-guanine phosphoribosyltransferase (*HPRT*) gene mutations in the peripheral blood lymphocytes (PBLs) of BD-exposed workers [5], while others failed to find increases in gene mutations [6–9]. For chromosome-level damage, a study in China [10] indicated positive induction of micronucleus (MN) in the PBLs of heavily exposed BD workers, but similar cytogenetic effects have not been indicated by several studies conducted on BD-exposed workers in Italy and the Czech Republic [11, 12]. Additional reports describe studies in Texas relating metabolic genotypes to* HPRT* mutation frequencies (MF), and some positive associations were found for mEH genotypes/phenotypes and* HPRT *MF [13, 14]. Significantly, further analysis on the original data of Czech workers has shown no BD effect revealed in the increased chromosomal aberrations among workers lacking the glutathione S-transferases T1 (*GSTT1*) gene compared to BD workers with the gene [11]. These results indicate the possibility of a different genetic background such as single nucleotide polymorphism (SNPs) that may play a critical role in BD's genotoxicity.

In recent years, BD epidemiological research has focused on clarifying the relationship between gene polymorphisms and the risk of mutagenicity and carcinogenicity. Two groups of environmental interactive genes: metabolic enzyme genes and DNA repair genes are the mostly studied ones. Metabolism is a focal point when evaluating the genotoxicity of BD in humans, because the epoxide metabolites of BD conduct the most DNA damage by bioalkylating DNA and forming adducts. Thus, the polymorphisms of metabolic genes (*CYP2E1*,* GST*s, and* mEH*) involved in BD metabolism were included in several studies to understand their relationship with BD genotoxicity. Epidemiological studies on BD-exposed workers indicated that many polymorphic loci of metabolic genes can impact the chromosomal damage induced by BD exposure [13–16]. In 2009, using cytokinesis-blocked micronucleus (CBMN) cytome assay, we found that BD-exposed workers exhibited increased frequencies of micronuclei (MNi) and nucleoplasmic bridges (NPBs) when compared to subjects in the control group. Further polymorphism analysis indicated that the BD-exposed workers carrying* CYP2E1* c1c2/c2c2 or* mEH* intermediate (I)/high (H) group had a significantly higher NPB frequency than those carrying* CYP2E1* c1c1 or the* mEH* low (S) group, respectively [17].

DNA repair is a universal process occurring in living cells. This process is responsible for the maintenance of the structural integrity of DNA in the face of damage arising from environmental insults, as well as from the normal metabolic processes. A study on the BD-exposed workers of Ningbo, China, examined the polymorphic variants in DNA repair genes, assuming that the ability of DNA repair that is different between individuals can modify the genotoxic effect of BD exposure [10]. The results showed that some SNP loci of* XRCC1* did impact the MNi frequencies of BD-exposed workers. XRCC1 is a protein essential to the repair of single strand breaks (SSBs) and base excision repair (BER) pathway [18]. XRCC1 acts as a scaffold protein and interacts with multiple DNA repair enzymes like poly (adenosine diphosphate-ribose) polymerases (*ADPRT*) and apurinic/apyrimidinic endonucleases (APE1). However, the research conducted on workers employed in tire plants of the Czech Republic did not find any significant association between genetic polymorphism of* XRCC1* exon 10 (*Arg399Gln*) and DNA damage biomarkers including chromosome aberrations and single strand breaks, where these workers were exposed to a variety of xenobiotics, the most prominent being BD and soot containing polycyclic aromatic hydrocarbons (PAHs) [19]. Thus, these inconsistent results indicated that the polymorphisms of* XRCC1* gene and associated DNA repair genes are worthy of further research to clarify their roles in BD-related genotoxicity. The DNA-repair enzyme O^6^-methylguanine-DNA methyltransferase (MGMT) is a key factor in the resistance to alkylating agents. The MGMT protein can rapidly reverse alkylation at the O^6^ position of guanine, thereby averting the formation of lethal cross-links [20].

Our group has conducted two studies concerning BD-exposed workers in 2002 and 2009, respectively. The 2002 study found that BD exposure did not statistically increase* HPRT* genes [6], while the following study conducted in 2009 showed significant chromosomal damage and positive associations with some metabolic genotypes in BD-exposed workers [17]. The aims of the present study were to determine whether the DNA repair genes (*XRCC1*,* MGMT*,* APE1*, and* ADPRT*) can modify the genetic instability induced by BD exposure in current BD workers.

## 2. Materials and Methods

### 2.1. Study Population

As described earlier [17], we conducted a 1 : 1 matched pair study at a petrochemical product company in the Nanjing area of China. Forty-five BD-exposed workers were paired with an appropriate control from the same plant and were engaged for the present study and matched by gender, smoking habits, and close age (±3 years). The control subjects were selected from employees working in the administrative office or circulating water workshop and showed no evidence of exposure to known genotoxic agents. Questionnaires for all the subjects were accompanied by regular physical examinations at the Yangzi Employee Hospital. Meanwhile, blood samples and urine samples were collected for further study. An informed consent was obtained from each subject at the start of this study.

### 2.2. Exposure Measurement

The sampling methods were described previously [17]. In brief, two air sampling ways were conducted for exposure assessment in the present study, namely, personal sampling and stationary sampling. Both sampling ways were performed by active air samplers, with Gilian-LFS3 (Sensidyne, Inc., USA) carried by workers for personal sampling and GilAir-5 (Sensidyne, Inc., USA) for stationary sampling. Nine BD workers and 4 administrative officers (as controls) carried active air samplers at a flow rate of 50 mL/min for 8 hours during the consecutive 3 sampling days for personal sampling, while 11 locations in the BD production workshop and 2 locations in the workplace of control group were chosen for stationary sampling, with 3 samples being collected at a pumping rate of 200 mL/min for 15 minutes in 3 consecutive days at each location. After sampling, each charcoal tube sample was sent for quantitative analysis, conducted according to the standard method (GBZ 160.39–2007).

### 2.3. Urinary Metabolite

Inhaled BD in the human body is metabolized through cytochrome P450-catalyzed oxidation processes to highly reactive epoxides. The epoxides can be hydrolyzed and conjugated with glutathione, leading to mercapturic acids which are excreted in urine. One of the major metabolites excreted in urine is N-acetyl-*S*-(3,4-dihydroxybutyl)-*L*-cysteine (DHBMA or M1). DHBMA was chosen as an internal exposure biomarker in the present study. A liquid chromatography tandem mass spectrometry (LC-MS/MS) was adopted to identify the urinary concentrations of DHBMA in both groups. Urine samples were collected after the work shift. After excluding unqualified urine samples according to the standard sampling and storage procedures (GBZ159-2004), 23 pairs of subjects were selected within the 45 pairs of included subjects to identify the concentrations of DHBMA. Briefly, urinary samples were thawed from −80°C to room temperature initially. Then, analytes were extracted from urine using solid phase extraction with a SAX column (Isolute ENV^+^) and quantified by LC-MS/MS analysis performed with an ion trap spectrometer. Ionization of the analytes was obtained by electrospray in negative mode and acquisition was performed in multiple-reaction monitoring mode, following the reaction* m/z*: 250.1 → 121 and 257.1 → 128.1 for DHMBA and DHBMA-D_7_. DHBMA and DHBMA-D_7_ standard materials were obtained from Toronto Research Chemicals (TRC), Ontario, Canada. The detection limit for the test substance was 10 *μ*g/L.

### 2.4. CB-MN Assay

The CBMN assay was performed according to standard methods described by Fenech [21]. This methodology was published previously [17]. In the present study, 0.5 mL of fresh blood was used to set up cultures for measuring. One thousand binucleated lymphocytes per subject were scored blindly by a single investigator for the presence of MNi, NPBs, and NBUDs. The MNi, NPBs, and NBUDs frequencies were the number of MNi, NPBs, and NBUDs observed per 1000 lymphocytes, expressed as a count per thousand (‰). The numbers of mono-, bi-, tri-, and tetranucleated cell in 500 lymphocytes were also scored for NDI calculation.

### 2.5. DNA Extraction and Genotyping

Genomic DNA was directly extracted from EDTA-anticoagulated whole blood using a wizard genomic DNA purification kit (Promega Corp., Madison, WI, USA) according to the manufacturer's instructions. PCR-RFLP was the main genotyping method employed. PCR-RFLP for* XRCC1*,* ADPRT, MGMT*,and* APE1 *SNP loci were performed under the following conditions: 95°C for 5 min, followed by 30 cycles of 94°C for 40 s, annealing for 20 s and 72°C for 35 s, and a final elongation step at 72°C for 10 min; the respective annealing temperature for each locus was as follows:* XRCC1 Arg194Trp *(58°C)*, XRCC1 Arg280His *(69.5°C),* XRCC1 Arg399Gln* (56°C),* XRCC1 T-77C* (58°C),* ADPRT Val762Ala* (58°C),* MGMT Leu84Phe* (58°C), and* APE1 Asp148Glu* (53°C). PCR products were digested with specific restriction enzymes that were recognized and cut either at the wild-type or variant sequence site. Primers and restricted endonucleases were shown in Table 1. The genotype results were regularly confirmed via direct DNA sequencing of the amplified fragments.

### 2.6. Statistical Methods

Normality tests showed that both exposure measurements and chromosomal damage data did not distribute normally; so Wilcoxon's rank sum test and Wilcoxon's signed rank test were applied to assess the differences between these two groups. Haplotype analysis was performed by PHASE 2.1 software. Poisson regression models as described by Wang et al. [10] were produced to quantify the relationship of chromosomal damage and the genotypes or diplotypes, estimated by the frequency ratio (FR) (FR = *e* ^*β*^, *e* = 2.71828,  *β*: regression coefficient) with 95% confidence intervals. FR was adjusted for age, sex, smoking status, and alcohol drinking in a multivariate Poisson regression analysis. For categorical variables, the FR indicated a proportional increase/decrease of the MN/NPB/NBUD frequency in a comparison group relative to the reference. Statistical analyses were performed using SAS 9.0 (SAS Institute Inc., USA).

## 3. Results

### 3.1. Baseline Information

The match up process resulted in 45 pairs of subjects. As described earlier [17], we found that the pairs were well matched for baseline information, such as gender (34 males and 11 females), age, and smoking habits (26 ex- or present smokers and 19 nonsmokers), with a mean age of 40.6 in both the BD-exposed group and the control group. All subjects are Han race Chinese.

### 3.2. Exposure Measurement

Environmental exposure data have been published [17]. Briefly, for personal sampling, each measurement was recorded as the 8 h time-weighted average (TWA), and, for the subjects' workshift, the average BD measurement for the exposed group (0.34 ± 0.61 p.p.m. or 0.75 ± 1.35 mg/m^3^) was significantly higher (*P* < 0.01) than that for the control group (0.04 ± 0.01 p.p.m. or 0.09 ± 0.02 mg/m^3^). For stationary sampling, the BD production plant had a mean concentration of 2.27 ± 3.33 p.p.m. or 5.02 ± 7.36 mg/m^3^. In the control administration office and the circulatory water plant, six measurements showed a low-level mean concentration of 0.84 ± 0.20 p.p.m. or 1.86 ± 0.44 mg/m^3^, which was significantly lower (*P* < 0.01) than that for the BD production plant.

### 3.3. Urinary Metabolite

Using LS-MS/MS methods, we identified urinary metabolites (DHBMA) concentrations of 23 pairs of subjects and found that the DHBMA median concentrations (as shown in Table 2) of BD-exposed workers were statistically higher than those for the controls.

### 3.4. Chromosomal Damage

The data on the frequencies of MNi, NPBs, NBUDs, and NDI have been published [17]. Briefly, the numbers of MNi (8.00 ± 3.78‰ versus 5.62 ± 2.41‰) and NPBs frequency (2.58 ± 2.79‰ versus 1.13 ± 1.34‰) in the BD-exposed workers were significantly higher (*P* < 0.01) and NDI (2.20 ± 0.14 versus 2.35 ± 0.27) was significantly lower (*P* < 0.01) in BD-exposed workers than in the control subjects, respectively. When age, gender, and smoking status factors were taken into consideration, the results showed that female workers had a borderline (*P* = 0.053) higher MNi frequency (9.45 ± 3.21‰) than the male workers (7.53 ± 3.87‰) in the BD-exposed group [17].

### 3.5. Distribution of Genotypes and Allele Frequencies

The allele frequencies for each single nucleotide polymorphism site are shown in Table 3. The genotype distributions at each locus were consistent with the Hardy-Weinberg equilibrium.

### 3.6. Polymorphism Analysis of DNA Repair Genes

A multivariate analysis using a backward stepwise selection of variables did not find that any SNP locus of* XRCC1*,* MGMT*,* ADPRT,* or* APE1* could impact chromosomal instability induced by BD exposure (data not shown).

To elucidate the relevance of* XRCC1* variants with chromosomal damage further, haplotypes among the four* XRCC1* polymorphisms (*XRCC1* -*77 C/T, Arg194Trp, Arg280His, *and* Arg399Gln*) were reconstructed. For BD-exposed workers, 14* XRCC1* (-77)-(194)-(280)-(399) diplotypes were identified in the analysis. The diplotype* TCGG-TCGG* which consists of the wild-type sequence in all loci was selected as the reference. Among these haplotype pairs, the rare diplotypes (<5% frequency) were analyzed as a group.

When* XRCC1* diplotypes were taken as categorical variables, after being adjusted by age, gender, occupational length, smoking, and drinking status in the multivariate Poisson regression model, we found that the workers carrying diplotypes* TCGA-CCGG* and* TCGG-TCGA* had statistically higher NBUDs frequencies than those who carried diplotype* TCGG-TCGG *among the BD-exposed workers (Table 4). The FRs associated with various diplotypes in all study subjects are presented in Table 5.

## 4. Discussion

There is sufficient evidence of potential carcinogenicity of BD in humans; thus, relevant occupational health organizations have reduced occupational exposure limits of BD. For example, in 1996, OSHA decided to lower permissible exposure limit (PEL) for BD in occupational-exposed workers from 1000 p.p.m. to 1 p.p.m. The data from two studies conducted by our group indicate that stationary sampling results in the present study decreased rapidly compared with that of 2002 study, and the personal sampling results were also below the OSHA limits. The decrease of the stationary sampling results in our studies, we believed, was due to the occupational protection effort made by the company administrators. However, in a comparison with short-time airborne environmental exposure samplings, the levels of chemical metabolites in the human body may reflect cumulative exposure status more accurately. Therefore, in a further study, we tested the internal exposure levels of BD in subjects as reflected by DHBMA. DHBMA and MHBMA are the main urinary metabolites of BD. It is claimed that DHBMA assay has the sensitivity to measure average BD exposure of 3 to 4 p.p.m. during the workday [22]. A study in Czech found that urinary DHBMA concentrations had increased in BD-exposed workers (median = 508 *μ*g/L) compared to controls (median = 355 *μ*g/L). And when BD-exposed workers were divided into >0.7 p.p.m. group (median = 2719 *μ*g/L) and <0.7 p.p.m. group (median = 669 *μ*g/L), the difference of urinary DHBMA concentrations were more significant [22]. In a study concerning butadiene-polymer workers in China [23], urinary DHBMA (M1) levels were substantially elevated in BD workers (median = 1.3 g M1/mg creatinine) too. A recent study in China (Ningbo) found that the average DHBMA levels of the BD-exposed workers were up to 617.82 ng/mL (or *μ*g/L) [24]. In the present study, urine DHBMA concentrations were also significantly higher in the BD-exposed workers (median = 122.54 *μ*g/L, range = 1080.10 *μ*g/L). The DHBMA levels of the present study were much lower than those of the Ningbo study, which was consistent with differences of airborne BD levels determined by TWA measurement between these two studies (0.75 ± 1.35 mg/m^3^ versus 2.40 ± 2.93 mg/m^3^). In conclusion, the present study demonstrated that the DHBMA urine metabolite concentrations could be a suitable biomarker for BD exposure.

In recent years, cytokinesis-block micronucleus cytome (CBMN Cyt) assay has evolved into a comprehensive method for measuring chromosome breakage, DNA misrepair, chromosome loss, nondisjunction, necrosis, apoptosis, and cytostasis [21]. Using CBMN cytome assay, our study shows that BD exposure could induce chromosomal damage (MNi and NPBs) in BD-exposed workers, which was consistent with two other studies in China [10, 16]. However, several studies have yielded negative results [6–9]. Individual differences especially genetic polymorphisms may contribute such inconsistent results. More and more attention has been paid to the polymorphisms of DNA repair genes when considering susceptibility factors involved in BD-induced genotoxicity in occupationally exposed workers. A recent study found that several polymorphic genes including* XRCC1* were associated with higher MN frequencies among BD-exposed workers [10]. Thus, it would seem likely that susceptible* XRCC1* might play a critical role in BD-related genotoxicity.

Eleven loci among 4 DNA repair genes were included for polymorphism analysis in the present study, but no single genotype of DNA repair genes could impact chromosomal damage in BD-exposed workers. However, in the further haplotype analysis, we found that those BD-exposed workers carrying diplotype* TCGA-CCGG *(*XRCC1* -*77-194-280-399*) and diplotype* TCGG-TCGA* exhibited more serious chromosomal damage (as reflected by NBUDs, compared to wild-type diplotype as a reference). We noticed that both of these two diplotypes shared one haplotype* TCGA*. Similarly, further haplotype analysis in a population of Ningbo area of China study found that BD-exposed workers with diplotype* TCGA-TCGA* had a higher MN frequency than the others [10]. Thus far, we have assumed that* TCGA* of* XRCC1* can be regarded as a risky haplotype for BD induced genetic damage. The XRCC1 protein was found to be responsible for the BER pathway. Evidence indicates that the VCM (vinyl chloride monomer) derivatives etheno-DNA adducts can be repaired through the BER pathway [25]. Meanwhile, the formation of DNA adducts by various BD metabolites is central to initiating the mutagenic process for BD exposure. XRCC1 protein may also play a role in the repair of BD-induced chromosomal damage through the BER pathway because of similar DNA adducts formation. Besides, Chinese hamster ovary cell lines (EM9 and EM-C11) with* XRCC1* mutant have revealed an unusually high frequency of sister chromatid exchange induced by alkylating agents, such cells reverting subsequently to the transfection of human* XRCC1* [26]. Other studies indicated that* XRCC1* haplotypes may be suitable to study the association of environmental factors and diseases. For example, Leng et al. reported that* XRCC1* haplotypes could impact chromosomal damage in Chinese coke oven workers [27]. The present study as well as the study in the Ningbo area of China indicates the possibility of* XRCC1* haplotypes used as a susceptibility biomarker to monitor BD occupational exposure in the future; however, extensive research is still needed on a larger sample size of population, especially outside China.

In our previous study, we found that MNi and NPBs frequencies differed significantly between BD-exposed workers and the controls, but no such response was observed for NBUDs frequencies [17]. When genetic susceptibility is taken into account in the present study, the polymorphisms of DNA repair genes (*XRCC1*) were found to be associated with NBUD frequencies in the BD-exposed workers. NBUD is regarded as a biomarker for the DNA repair process and is associated with molecular events, such as nuclear elimination of amplified DNA and/or DNA repair DNA-protein complexes [21]. Nuclear budding and micronucleation have often been seen as part of a p53-dependent DNA repair mechanism for the removal of promiscuous DNA [28]. Dutra et al. reported NBUDs highly induced in cultured fibroblasts taken from transgenic mice with a DNA repair deficiency and a Trp53 deficiency [29]. The present results provide some evidence that the induction of NBUDs is related to DNA repair genes in human epidemiological studies. However, whether NBUDs are a mechanism to eliminate excess chromosomes in a hypothesised process known as aneuploidy rescue remains unclear [30]. And the mechanism in detail for how the* XRCC1* gene was involved in this process still needs more studies. The polymorphisms of the metabolic genes and DNA repair genes associated with NPBs or NBUDs frequencies in BD-exposed workers highlight the importance of the newly developed biomarkers for the CBMN cytome assay in occupational exposure biomonitoring.

In conclusion, our study indicates that occupational exposure to BD may impact chromosomal instability (MNi and NPBs) of those workers. The results of polymorphism analysis and haplotype analysis suggest that the diplotype of* XRCC1* (*TCGA-CCGG *and* TCGG-TCGA*) elevated the NBUD frequencies among BD-exposed workers. However, additional studies are recommended to validate these findings in larger population in the future.

## Figures and Tables

**Table 1:** PCR primers and restricted endonucleases for each of DNA repair genes in genotyping process.

Gene	Primers		PCR method	Restricted endonucleases
	Forward	Reverse		
*XRCC1 194 *	5′-GCCCCGTCCCAGGTA-3′	5′-AGCCCCAAGACCCTTTCACT-3′	RFLP	MspI
*XRCC1 280 *	5′-TGGGGCCTGGATTGCTGGGTCTG-3′	5′-CAGCACCACTACCACACCCTGAAGG-3′	RFLP	RsaI
*XRCC1 399 *	5′-TTGTGCTTTCTCTGTGTCCA-3′	5′-TCCTCCAGCCTTTTCTGATA-3′	RFLP	MspI
*XRCC1 -77 *	5′-GAGGAAACGCTCGTTGCTAAG-3′	5′-TCCTCATTAATTCCC TCACGTC-3′	RFLP	BsrBI
*ADPRT 762 *	5′-TTTTGCTCCTCCAGGCCAAcG-3′	5′-CCTGACCCTGTTACCTTAATGTCAGTTTT-3′	RFLP	Hinf1
*MGMT 84 *	5′-AAGAGTTCCCCGTGCCGAC-3′	5′-GCCAAACGCTGCCTCTGT-3′	RFLP	HinfI
*APE1 148 *	5′-CTGTTTCATTTCTATAGGCTA-3′	5′-AGGAACTTGCGAAA GGCTTC-3′	RFLP	Bfa1

**Table 2:** Urinary metabolites of BD-exposed workers and controls (23 pairs).

	Exposure (p.p.b.)				Control (p.p.b.)			
	Range	Selected percentile			Range	Selected percentile		
		25th	50th (Median)	75th		25th	50th (Median)	75th
DHBMA	<10~1080.10	56.10	122.54^*^	199.23	<10~214.76	<10	10.70	61.09

^*∗*^Compared between exposure group and matched controls, *P* < 0.01; p.p.b.: *μ*g/L.

**Table 3:** Distribution of genotypes and allele frequencies among 1,3-butadiene- (BD-) exposed workers.

SNPs	Genotypes	*N*	Rate%	Frequency
*XRCC1 -77 *	TT	30	66.7	T: 0.83
	CT	15	33.3	C: 0.27
	CC	0		

*XRCC1 194 *	CC	37	82.2	C: 0.91
	CT	8	17.8	T: 0.09
	TT	0		

*XRCC1 280 *	GG	36	80.0	G: 0.88
	GA	7	15.6	A: 0.12
	AA	2	4.4	

*XRCC1 399 *	GG	27	60.0	G: 0.76
	GA	14	31.1	A: 0.24
	AA	4	8.9	

*ADPRT 762 *	TT	15	33.3	T: 0.56
	CT	20	44.4	C: 0.44
	CC	10	22.3	

*MGMT 84 *	CC	32	71.1	C: 0.84
	CT	12	26.7	T: 0.16
	TT	1	2.2	

*APE1 148 *	TT	11	24.4	T: 0.54
	GT	27	60.0	G: 0.46
	GG	7	15.6	

**Table 4:** Chromosomal damage between diplotypes of *XRCC1* in BD-exposed workers.

Diplotypes	*N* (%)	MN (‰)	NPB (‰)	NBUD (‰)	NDI
*TCGG-TCGG *	9 (20.0)	8.89 ± 4.40	2.44 ± 2.01	1.89 ± 1.27	2.21 ± 0.17
*TCGG-CCGG *	7 (15.6)	6.14 ± 3.13	2.00 ± 2.38	3.43 ± 1.98	2.11 ± 0.09
*TCGG-TCAG *	5 (11.1)	7.00 ± 2.24	3.60 ± 4.04	2.60 ± 1.67	2.22 ± 0.14
*TCGA-CCGG *	5 (11.1)	8.50 ± 2.89	4.25 ± 2.87	4.25 ± 2.06^*^	2.07 ± 0.11
*TCGG-TCGA *	4 (8.9)	11.60 ± 4.61	2.40 ± 0.55	5.80 ± 3.56^*^	2.25 ± 0.05
Others	15 (33.3)	7.33 ± 3.54	2.20 ± 3.43	2.73 ± 2.19	2.25 ± 0.13

The diplotype is defined as the allele present at positions* −77 (C/T)*, *194 (C/T), 280 (G/A)*, and *399 (G/A)*, respectively.

^*^As seen in Table 5, *P* < 0.05.

**Table 5:** Association between diplotypes of *XRCC1* and the frequency of nucleus buds (NBUDs).

Name	*β*	95% CI		*X* ^2^	*P*	Adjusted FR^a^
		Low	Upper			(95% CI)
Intercept	0.6047	−0.3894	1.5988	1.42	0.2332	
Gender (female)	0.3118	−0.2169	0.8405	1.34	0.2478	1.37 (0.81–2.32)
Age (*⩽*40)	0.1287	−0.4292	0.6865	0.20	0.6512	1.14 (0.65–1.99)
OL	−0.1447	−0.6655	0.3760	0.30	0.5859	0.87 (0.51–1.46)
Smoking (no)	−0.0894	−0.5808	0.4021	0.13	0.7215	0.91 (0.56–1.49)
Drinking (no)	−0.3682	−0.8256	0.0891	2.49	0.1145	0.69 (0.44–1.09)
*TCGG-TCGG *	—	—	—	—	—	1
*TCGG-CCGG *	0.5219	−0.1652	1.2091	2.22	0.1366	1.69 (0.85–3.35)
*TCGG-TCAG *	0.2441	−0.4885	0.9767	0.43	0.5137	1.28 (0.61–2.66)
*TCGA-CCGG *	0.7404	0.0274	1.4534	4.14	0.0418	2.10 (1.03–4.28)^*^
*TCGG-TCGA *	1.0101	0.3804	1.6399	9.88	0.0017	2.75 (1.46–5.15)^*^
Others	0.3530	−0.2696	0.9757	1.23	0.2665	1.42 (0.76–2.65)

The diplotype is defined as the allele present at positions *−77 (C/T), 194 (C/T), 280 (G/A)*,and *399 (G/A)*, respectively.

Others: grouping of all diplotypes with <5% frequency.

^a^Multiple Poisson regression: FR adjusted by age, gender, smoking, and occupational longevity (OL).

^*^ *P* < 0.05.
